# Supplementary material for: A Novel HGF/SF Receptor (MET) Agonist Transiently Delays the Disease Progression in an Amyotrophic Lateral Sclerosis Mouse Model by Promoting Neuronal Survival and Dampening the Immune Dysregulation
Source: Int J Mol Sci. 2020 Nov 12;21(22):8542. doi: 10.3390/ijms21228542 (PMC7696450; doi:10.3390/ijms21228542)
Supplement: Supplementary file 1 [file ijms-21-08542-s001.zip › ijms-998513-supplementary.docx]

Supplementary Materials

A Novel HGF/SF Receptor (MET) Agonist Transiently

A Novel HGF/SF Receptor (MET) Agonist Transiently Delays the Disease Progression in an Amyotrophic Lateral Sclerosis Mouse Model by Promoting Neuronal Survival and Dampening the Immune Dysregulation

Antonio Vallarola ^1,†^, Massimo Tortarolo ^1,†^, Roberta De Gioia ^1^, Luisa Iamele ^2^, Hugo de Jonge ^2^, Giovanni de Nola ^3^, Enrica Bovio ^2^, Laura Pasetto ^4^, Valentina Bonetto ^4^, Mattia Freschi ^1^,
Caterina Bendotti ^1,^* and Ermanno Gherardi ^2^

^1^ Laboratory of Molecular Neurobiology, Department of Neuroscience, Istituto di Ricerche Farmacologiche Mario Negri IRCCS, 20156, Milan, Italy; antonio.vallarola@unimore.it (A.V.);
massimo.tortarolo@marionegri.it (M.T.); robertadegioia1989@gmail.com (R.D.G.); mattia.freschi14@gmail.com (M.F.)

^2^ Immunology and General Pathology Unit, Department of Molecular Medicine, Università di Pavia, 27100, Pavia, Italy; luisa.iamele@unipv.it (L.I.); hugo.dejonge@unipv.it (H.d.J.); enrica.bovio@unipv.it (E.B.); egherard@unipv.it (E.G.)

^3^ Department of Neurobiology, Harvard Medical School, Boston, MA 02115, USA; gdenola@crystal.harvard.edu

^4^ Laboratory of Translational Biomarkers, Department of Biochemistry and Molecular Pharmacology, Istituto di Ricerche Farmacologiche Mario Negri IRCCS, 20156 Milan, Italy; laura.pasetto@marionegri.it (L.P.); valentina.bonetto@marionegri.it (V.B.)

***** Correspondence: caterina.bendotti@marionegri.it

† These authors contributed equally to this work.

Received: 28 October 2020; Accepted: 10 November 2020; Published: date

**Supplementary Materials:**


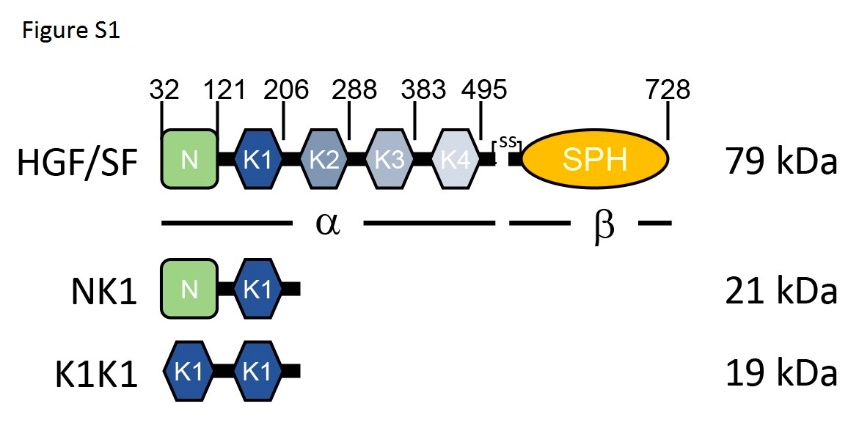


**Figure S1.** Schematic representation of the domain structure of the recombinant proteins used in this study. HGF/SF has a complex multi-domain structure comprising six domains and two disulphide-bonded chains. NK1 is a natural splice-variant of HGF/SF with reduced agonistic activity. The design of recombinant K1K1 is based on extensive functional and structural studies and is comprised of two covalently-linked HGF/SF kringle 1 domains providing two MET receptor binding sites in one molecule.


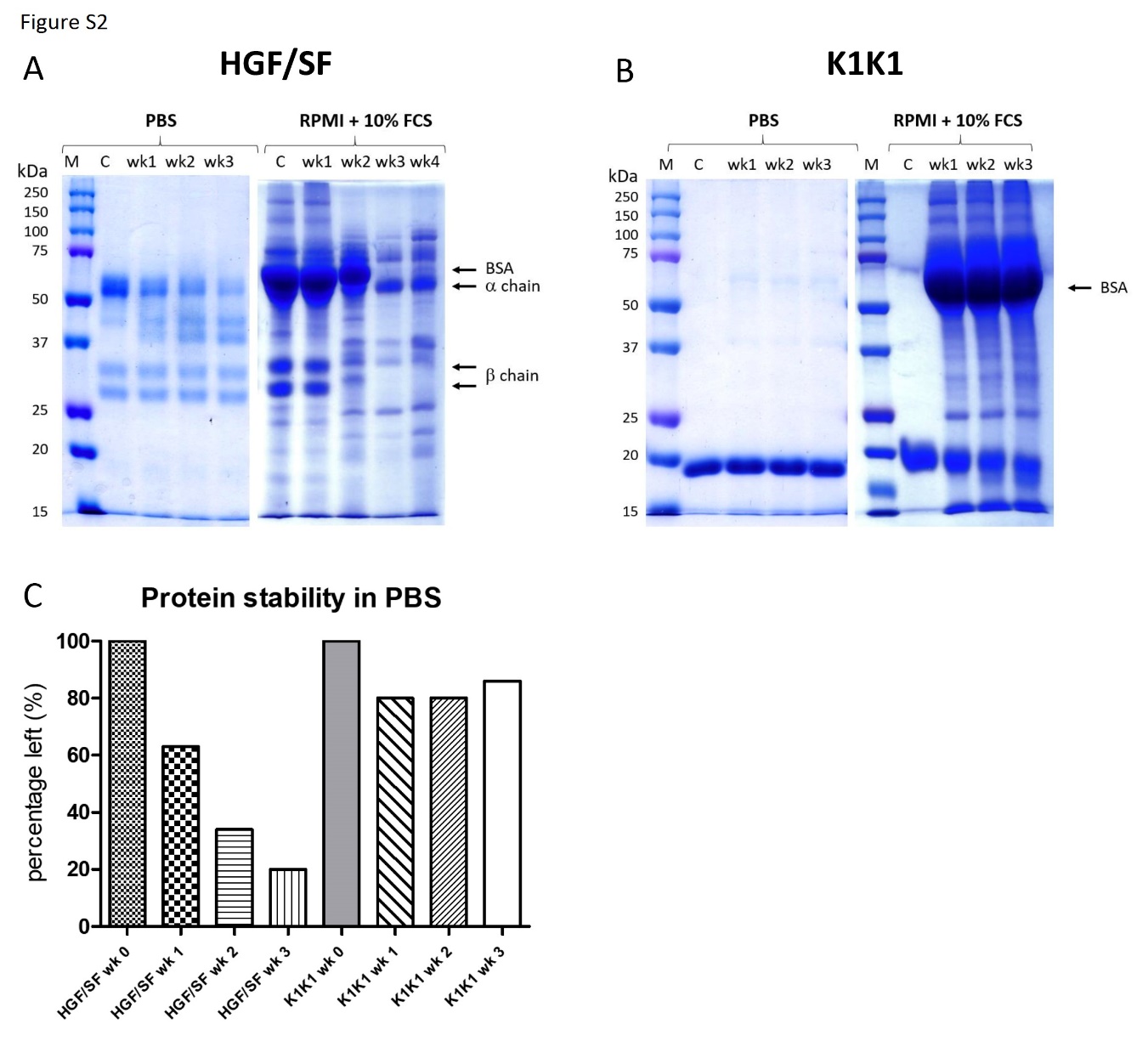


**Figure S2.** Coomassie staining and Western blot after SDS-PAGE showing stability of HGF/SF and K1K1 samples in PBS and in RPMI containing 10% FCS after different incubation periods at 37 °C. (**A**) Coomassie stained gel with HGF/SF samples incubated in PBS or RPMI+10%FCS taken before (“C”) and after subsequent weeks (wk1, wk2, wk3, wk4) of incubation at 37°C. Arrows indicate the molecular weight at which bovine serum albumin (BSA), the alpha chain and the beta chain (bottom) run on gel. Doublets are visible for the beta chain due to variation in glycosylation. 5 ug was loaded per lane. (**B**) Coomassie stained gel with K1K1 samples incubated in PBS or in RPMI+10%FCS after subsequent weeks (wk1, wk2, wk3) of incubation at 37°C. In the lane designated with “C” purified K1K1 without additional buffer or RPMI was loaded. 3.5 ug was loaded per lane. (**C**) Normalised fluorescence at 330 and 350 nm (so called “brightness values”) at different time points of HGF/SF and K1K1 samples in PBS measured with the NanoTemper Tycho NT.6. Initial measured brightness was set at 100% and the subsequent reduction in brightness is calculated as a percentage of these initial values.


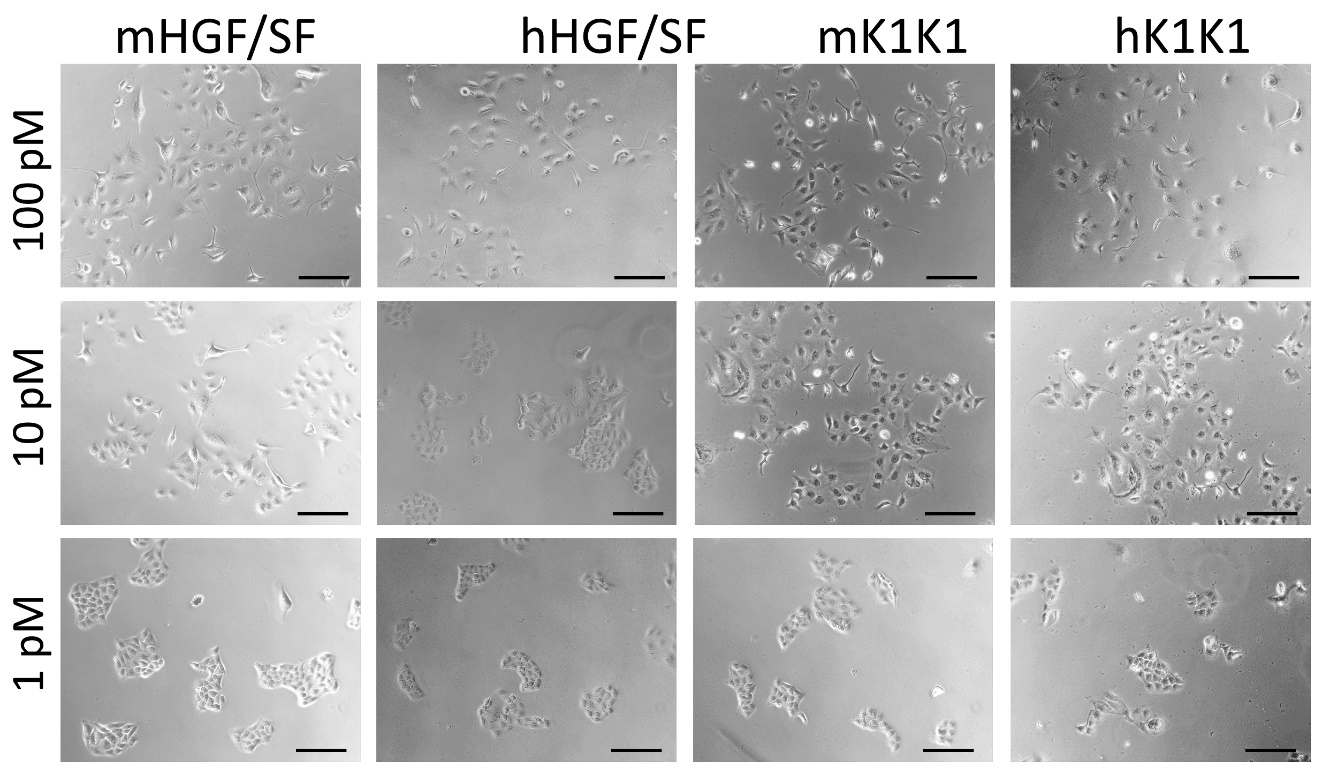


**Figure S3.** Comparison of human and mouse variants of HGF/SF and K1K1 in Madin-Darby Canine Kidney Cell scatter assay. Three different concentrations of mouse HGF/SF (mHGF/SF), human HGF/SF (hHGF/SF), mouse K1K1 (mK1K1), and human K1K1 (hK1K1) were tested for their potency to induce scattering. At 1 pM concentration, the cells form tight colonies as all proteins induce little to no scattering activity. At 10 pM and 100 pM the colonies are disrupted and most cells are scattered. Scale bars: 200 μm.


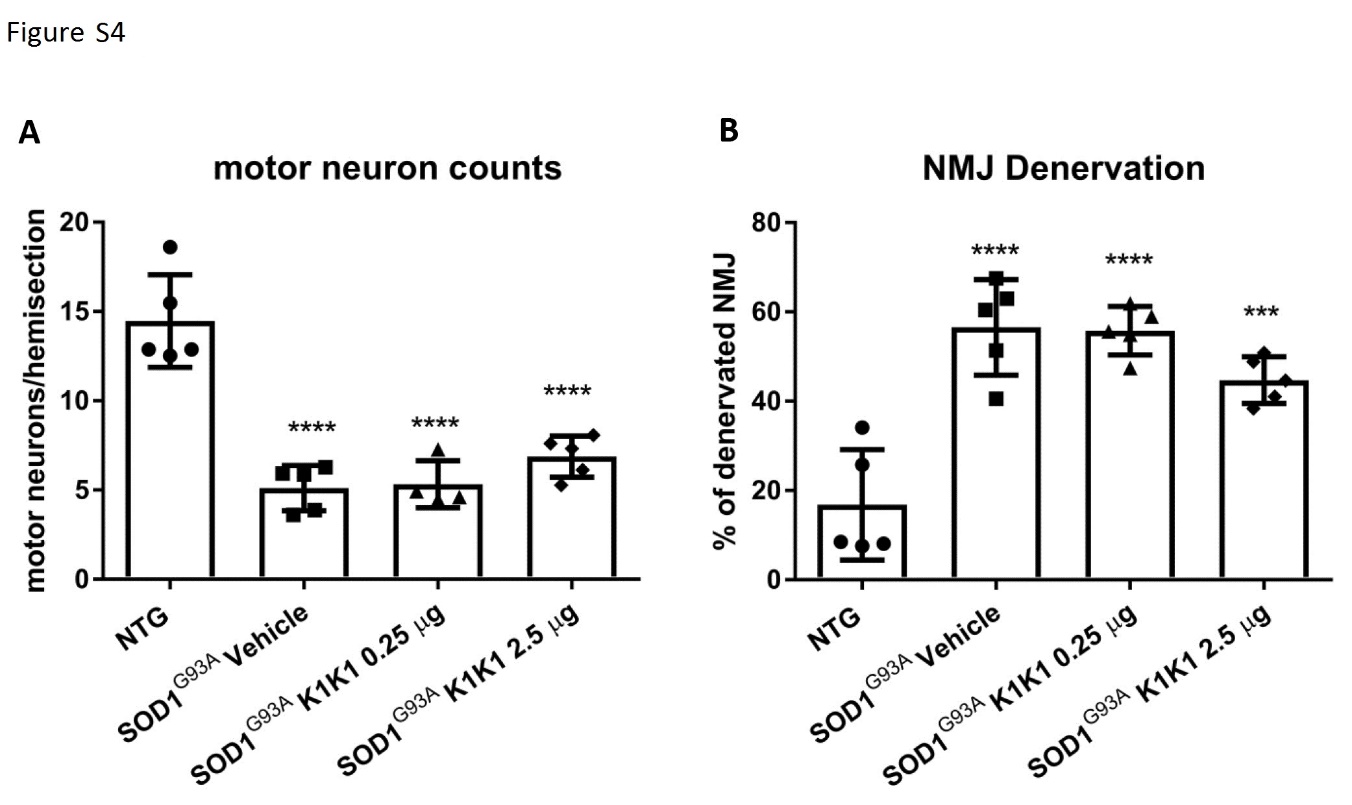


**Figure S4.** Lower doses of K1K1 do not protects motor neurons in the lumbar spinal cord and NMJ in tibialis anterior muscle (TAM) of SOD1G93A mice. (A) Quantification of motor neurons with cell body area > 400 μm2 in LSC. At symptomatic stage of the disease, MNs were decreased in transgenic mice compared to NTG but no difference was found in K1K1 (0.25 and 2.5 μg) treated mice. (B) Denervation of NMJ was higher in TAM of transgenic mice treated with vehicle but the treatment with K1K1 (0.25 and 2.5 μg) did no reduce this effect. Data are expressed as mean ±SEM, (*n* = 5 animals per group). Data were analysed with One-way ANOVA followed by post-hoc Fisher’s LSD. *** *p* < 0.01, **** *p* < 0.001.


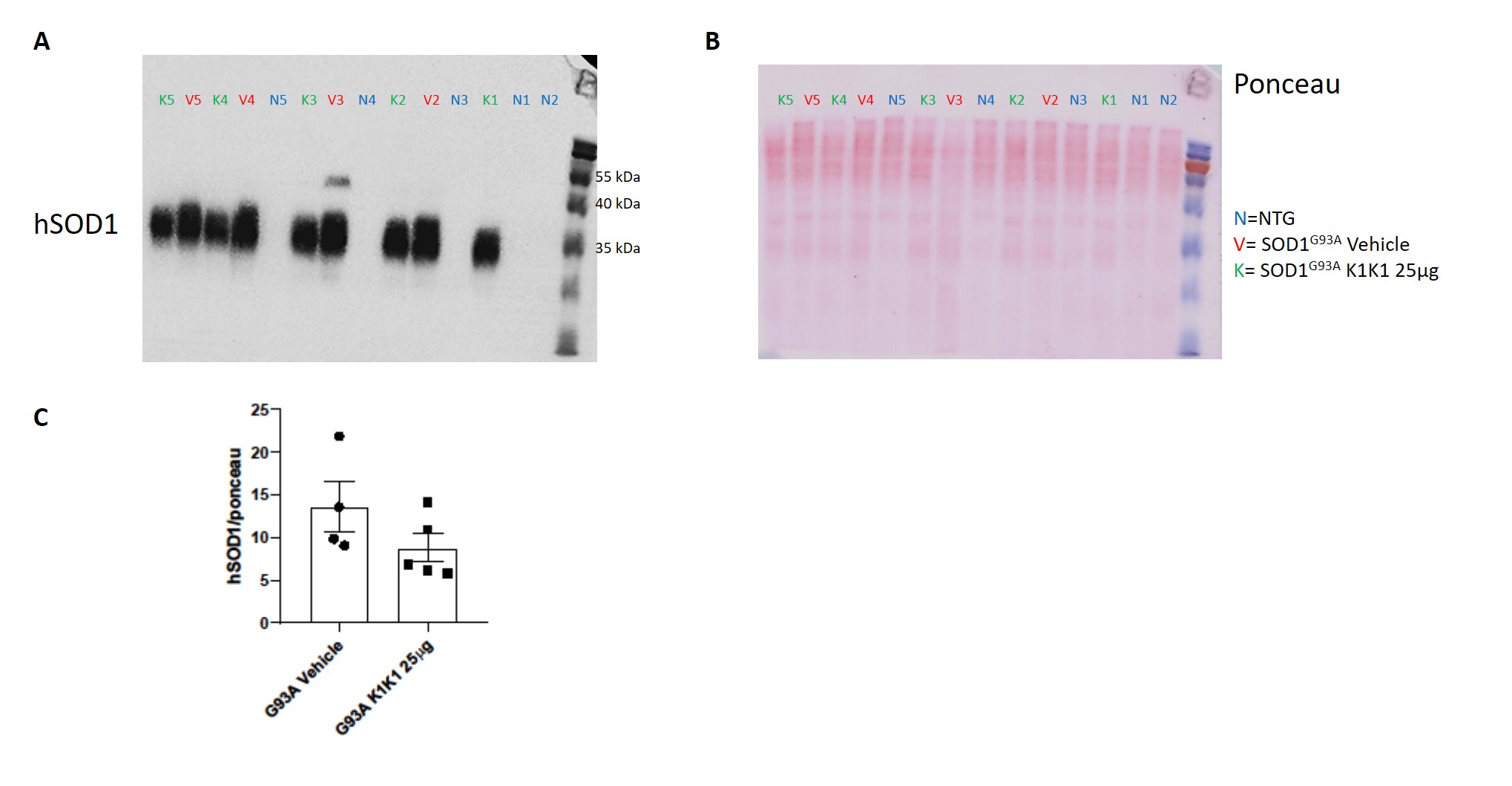


**Figure S5.** Levels of mutant human SOD1 in lumbar spinal cord are not modified by the K1K1 treatment. (**A**) Immunoblot of the human SOD1 and (**B**) corresponding membrane stained with red Ponceau staining from the ventral portion of the lumbar spinal cord. (**C**) Relative quantification of the immunoreactive bands normalized to the total amount of protein detected by red Ponceau. Bars represent mean ± SEM of ratio. Data were statistically analysed using a Student’s t-test.


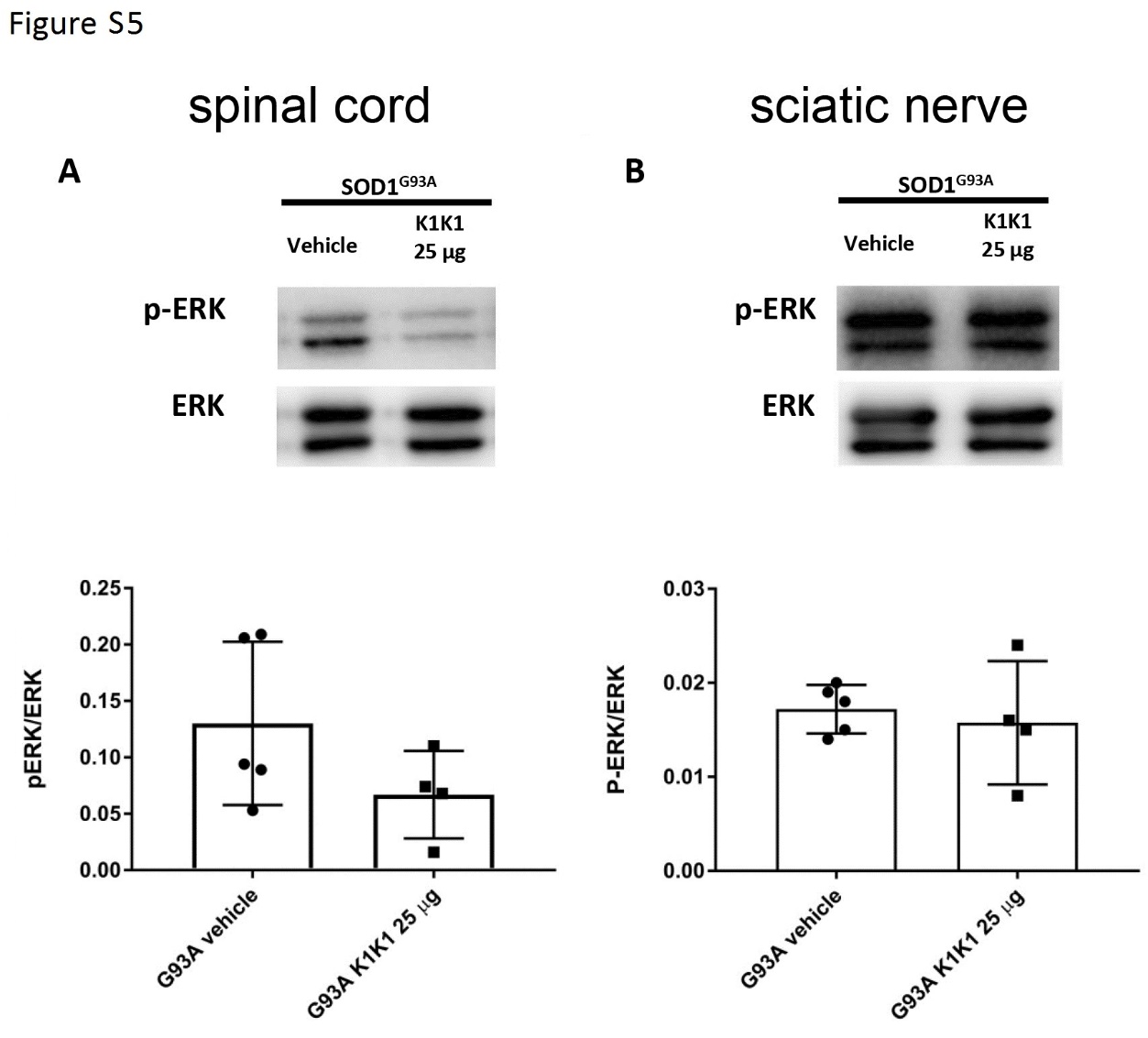


**Figure S6.** Prolonged treatment with 25 μg K1K1 loses effectiveness in activating p-ERK in lumbar spinal cord and sciatic nerve of SOD1G93A mice. Representative immunoblots of p-ERK/ERK and relative quantification performed on the ventral portion of lumbar spinal cord (A) and sciatic nerve (B) of transgenic SOD1G93A mice treated with vehicle or 25 μg K1K1 until death. Bars represent mean ± SEM of ratio. All data were statistically analysed using a Student’s t-test.

**
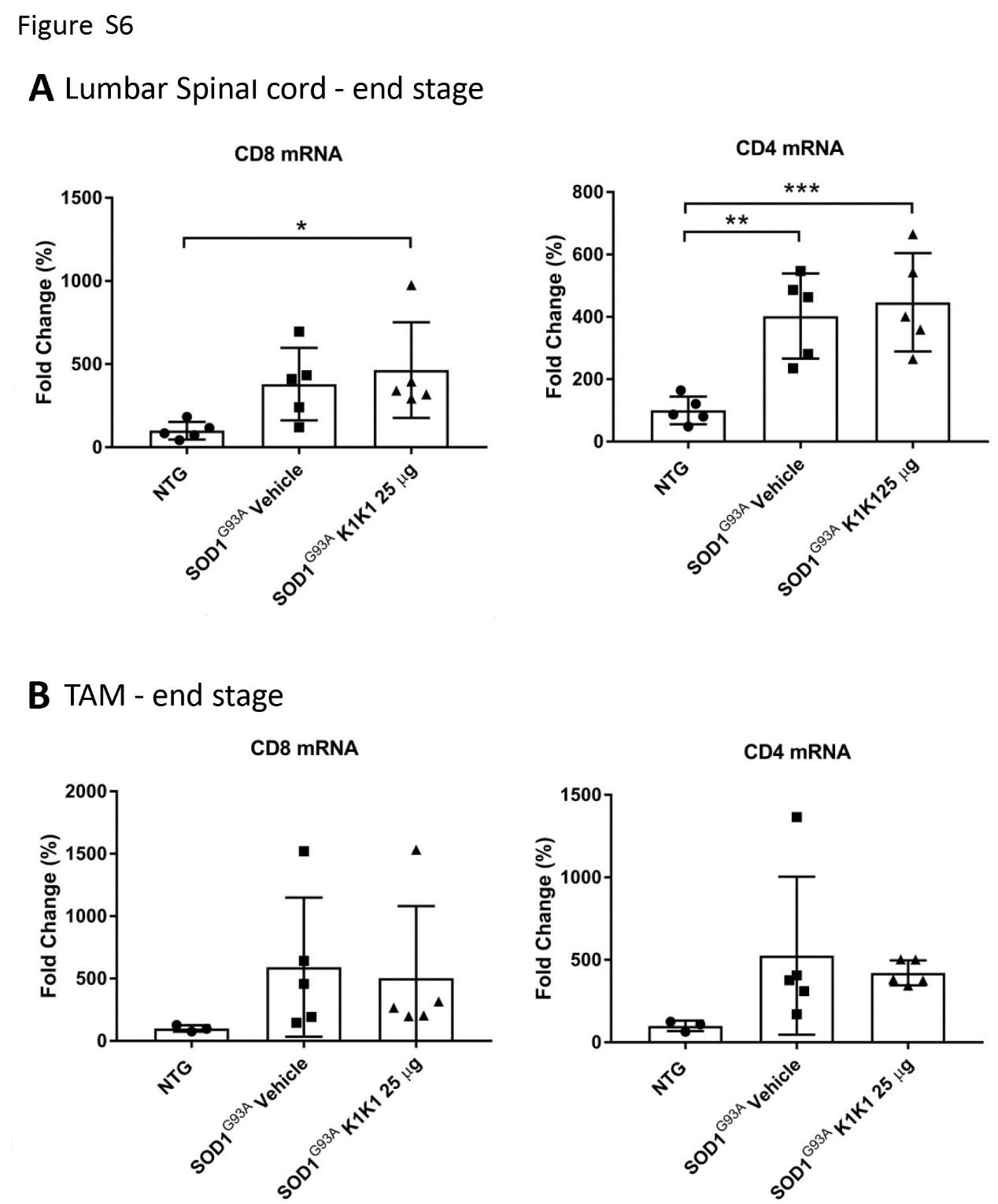
**

**Figure S7.** Prolonged treatment with K1K1 loses effectiveness in modulating the infiltration of T cells in the spinal cord and skeletal muscle of SOD1G93A mice. At the final stage of disease, treatment with K1K1 was no longer able to counteract the increase of CD8+ or CD4+ T cells recruitment in lumbar spinal cord (**A**) and in Tibialis Anterior Muscle (**B**). Data are expressed as the mean ± SEM (*n* = 3-5 mice per group). All data were statistically analysed using One-way ANOVA followed by post hoc Fisher’s LSD. * *p* < 0.05, ** *p* < 0.01, *** *p* < 0.001, n.s. = not significant.
